# Anterior Cingulate Cortex Mediates State-Dependent Prioritization of Distressed Conspecifics

**DOI:** 10.3390/brainsci16060658

**Published:** 2026-06-22

**Authors:** Hongyu Cao, Yang Zhou, Saifeng Zhao, Chenyi Guo, Xiao-Dan Yu, Wenhui Xiao, Ge Hu, Lianyan Huang, Boxing Li, Xiao Min Zhang

**Affiliations:** 1 School of Biomedical Engineering, Southern Medical University, Guangzhou 510515, China; 2 Zhongshan School of Medicine, Sun Yat-sen University, Guangzhou 510080, China

**Keywords:** social discrimination, anterior cingulate cortex, social behavior, optogenetics, fiber photometry

## Abstract

**Highlights:**

**What are the main findings?**

- Naïve and relieved mice rapidly prioritize distressed peers in complex environments.
- Acute stress experience induces profound reduced discrimination among demonstrators in observer mice.
- ACC excitatory activity causally dictates innate prioritization of stressed peers.

**What are the implications of the main findings?**

- Internal stress impairs social cognition by actively suppressing ACC neural dynamics.
- Targeted ACC activation restores stress-induced reduction in discrimination in complex social context.

**Abstract:**

**Background/Objectives:** Emotion recognition is a fundamental component of mammalian social cognition, enabling animals such as rodents to detect the emotional states of conspecifics and guide prosocial or avoidance behaviors. However, natural social landscapes are highly complex and multi-targeted. Furthermore, it remains unknown how an observer’s own internal emotional state dictates social target prioritization in these dynamic environments. **Methods:** We used a novel multi-target social paradigm wherein observer mice subjected to naïve, positive (relieved), or negative (acute stress) experiences, interacting simultaneously with demonstrator mice in neutral, relieved, or stressed states. We utilized *in vivo* fiber photometry to record anterior cingulate cortex (ACC) calcium dynamics during these complex interactions, and employed bidirectional optogenetics to establish causal neural mechanisms. **Results:** Naïve and relieved observer mice exhibited a rapid, innate behavioral prioritization of acutely stressed conspecifics. Conversely, an internal state of acute stress experience in the observer completely abolished this early-stage discrimination. Fiber photometry revealed that ACC excitatory neuronal activity robustly encodes the prioritization of stressed conspecifics, a neural signature that is fundamentally suppressed in stressed observers. Optogenetic inhibition of the ACC abolished innate social preference in naïve and relieved mice, whereas targeted ACC activation successfully overrode internal stress to restore social discrimination in stressed observers. **Conclusions:** Acute negative internal states profoundly suppress social discrimination ability. The ACC acts as a state-dependent gatekeeper to dictate social prioritization.

## 1. Introduction

The ability to recognize and respond to the emotional states of others is a cornerstone of social cognition. In rodents, this capacity is well documented; they can readily detect affective changes in conspecifics and exhibit targeted prosocial behaviors toward both stressed and relieved peers [1,2,3,4,5,6,7]. However, our current understanding stems largely from simplified, dyadic behavioral paradigms [8,9,10]. These settings inherently strip away the complexity of natural social environments, where animals are continuously confronted with multiple individuals in varying emotional states. To successfully navigate this complex social landscape, an animal must engage in “social prioritization”—the ability to weigh competing social stimuli and selectively allocate attention to specific peers. How an observer makes these critical prioritization decisions is not well understood. Crucially, it remains an open question whether an observer’s own internal state [11,12,13,14,15], shaped by recent experiences of stress or relief, overrides their natural social instincts to change this priority.

Integrating an animal’s internal emotional state with the dynamic demands of a complex social landscape requires a higher-order neural hub. The anterior cingulate cortex (ACC) has emerged as a prime candidate for this role. Positioned as a key node within the limbic system, the ACC shares dense connections with prefrontal, parietal, and subcortical regions governing valuation and action selection [16]. Functionally, it is broadly implicated in emotion regulation, pain perception, and the processing of observational distress [17,18,19,20,21,22,23,24]. For instance, human neuroimaging studies consistently demonstrate robust ACC activation when individuals observe fear in others [25,26]. Paralleling these human data, recent *in vivo* calcium imaging in rodents has revealed that transient ACC activation correlates strongly with prosocial approach behaviors, and that distinct neuronal ensembles respond specifically when mice observe conspecifics in pain or fear [27,28,29]. Despite these insights, a critical gap remains: it is currently unknown whether the ACC actively computes the relative emotional valences of multiple interacting peers in a complex environment, or whether its evaluative function is causally gated by the observer’s own internal affective state.

To address these critical gaps, we utilized a multi-target social paradigm based on recently established models [30]. Here, we demonstrate that naïve and relieved mice exhibit a rapid innate social prioritization of acutely stressed conspecifics, whereas acute stress in the observer induces a profound, state-dependent loss of social discrimination. To uncover the real-time neural correlates of this behavior, we utilized *in vivo* fiber photometry [31,32,33] to monitor population calcium dynamics in the ACC. We reveal that ACC calcium dynamics robustly encode the prioritization of stressed conspecifics, but this neural signature is entirely suppressed in stressed observers. Finally, through targeted optogenetic manipulation [34], we establish that ACC excitatory activity is causally required for rapid social discrimination, and that artificial activation of the ACC is sufficient to override the effects of internal stress and restore normal social prioritization.

## 2. Materials and Methods

### 2.1. Animals

All procedures were approved by the Institutional Animal Care and Use Committee (IACUC) of Sun Yat-sen University. Adult mice (6–8 weeks) were housed under a 12 h light/dark schedule with food and water available ad libitum. Wild-type (C57BL/6J) and transgenic (Thy1-GCaMP6s) mice were obtained from the Laboratory Animal Center of Sun Yat-sen University (Guangzhou, China) and the Jackson Laboratory (Bar Harbor, ME, USA, JAX:024275), respectively.

### 2.2. Multi-Target Social Paradigm

#### 2.2.1. Preparation of Demonstrator and Observer Mice

To investigate the impact of prior emotional experience on social preference, we established three distinct affective states in both demonstrator and observer mice: Naïve (neutral), Relieved (positive), and Stressed (negative).

**Demonstrator Mice**

Demonstrator mice were prepared immediately prior to the social interaction assay.

Naïve Demonstrators (ND): Mice remained undisturbed in their home cages until the commencement of the test.

Relieved Demonstrators (RD): Mice were subjected to a 24 h water deprivation period, followed by 30 min of *ad libitum* access to a sucrose solution immediately before the assay. This protocol induces a state of positive affective contrast (relief).

Stressed Demonstrators (SD): Mice were subjected to 15 min of acute physical restraint in a ventilated cylindrical tube immediately preceding the behavioral test.

**Observer Mice**

To distinguish the influence of prior emotional history from acute physiological arousal, observer mice underwent affective induction 24 h before the social preference test.

Naïve Observers (N-OB): Baseline control mice that underwent no prior manipulation.

Relieved Observers (R-OB): Mice underwent the relief protocol (24 h water deprivation followed by 30 min sucrose access) 24 h prior to the social assay.

Stressed Observers (S-OB): Mice were subjected to 15 min of acute restraint stress 24 h before the social assay.

#### 2.2.2. Multi-Target Social Behavioral Paradigm

The testing apparatus consisted of a square open field without a top cover (34 × 34 × 23 cm^3^), surrounded by black walls, with a white bottom plate and a camera installed on the top for continuous video recording.

Three distinct demonstrator mice were simultaneously introduced in three corners of the experimental box, respectively. At the beginning of the experiment, an observer mouse was gently placed in the center of the open field, facing away from the demonstrator mice, to minimize the mice’s early preference. The mouse was allowed to freely explore for 4 min, during which their behavioral activities were recorded throughout the process. After the test, the observer mouse returned to its original cage, and the box was thoroughly cleaned with 75% ethanol to remove odor cues. In this experiment, we used 13 mice as N-OB, 14 mice as R-OB, 11 mice as S-OB.

For behavioral analysis, the total duration of sniffing directed toward each demonstrator was quantified. To account for inter-individual variability in baseline exploration, sniffing behavior was normalized; the time the observer spent sniffing the SD or RD was normalized to the time spent sniffing the naïve demonstrator.

### 2.3. Open Field Test

Locomotor activity and anxiety-like behaviors were assessed using the open field test. A total of 15 observer mice were used: N-OB 5 mice, R-OB 5 mice, and S-OB 5 mice. The apparatus consisted of a square plastic arena (34 × 34 × 23 cm^3^), placed in a dimly lit and sound-attenuated room. Each mouse was gently placed in the center of the arena and allowed to explore freely for 10 min. The behavior was recorded by a video camera mounted above the center of the arena.

### 2.4. Three-Chamber Social Test

Mice were first habituated for 5 min in an empty (60 × 40 × 20 cm^3^) rectangular white acrylic box arena divided into three interconnected chambers (left, center, and right). 5 mice from each observer group were tested. Sociability was evaluated during a second 5 min period in which the subject could interact either with an empty wire cup (Empty) or a wire cup containing a stranger conspecific (Mouse 1). The interaction time was determined by measuring the time the subject mouse spent sniffing or climbing upon either the empty cup or the cup containing the stranger mouse. The position of the empty cup/stranger mouse in the left or right chamber during the sociability period was counterbalanced between trials to avoid bias. Preference for social novelty was assayed in a third 5 min period by introducing a second stranger mouse (Mouse 2) into the previously empty wire cup. Between trials, the apparatus was thoroughly cleaned with 70% ethanol to remove olfactory cues. Sociability index = (T_mouse1_ − T_empty_)/(T_mouse1_ + T_empty_), social novelty index = (T_mouse2_ − T_mouse1_)/(T_mouse2_ + T_mouse1_).

### 2.5. Serum Corticosterone Quantification

Blood samples were collected from the 15 mice from the 3 observer groups via the retro-orbital venous plexus. Samples were allowed to clot at room temperature (25–28 °C) for 2 h and centrifuged at 2000 rpm for 20 min at 4 °C to obtain serum. Serum corticosterone levels were measured using a corticosterone ELISA kit (AMOYLUNCHANGSHUOBIOTECH Co., Ltd., Xiamen, China, cat. no. WLCSJZF20546) according to the manufacturer’s instructions.

Briefly, serum samples were diluted 1:5 prior to analysis. All reagents and samples were equilibrated to room temperature (25–28 °C) for 1 h and processed using the ELISA kit according to standard procedures. Absorbance was measured at 450 nm using a TECAN Sunrise microplate reader (TECAN, Grödig, Austria), and corticosterone concentrations were calculated from a standard curve generated using CurveExpert software (v 1.4, Hyams Development, Huntsville, AL, USA).

### 2.6. Fiber Optic Implantation Surgery

The Thy1-GCaMP6s mice were used in the experiment. At least 10 days prior to behavioral testing, the mice underwent stereotactic surgery to implant fiber optic catheters. Before the surgery, the mice were anesthetized with intraperitoneal injection of Avertin (250 mg/kg), and mounted on a stereotaxic instrument (Nanjing ThinkerTech, Nanjing, China).

Based on the mouse brain atlas (Franklin & Paxinos, 3rd ed.), a cranial hole with a diameter of approximately 0.8 mm was drilled in the ACC brain region (AP: 0.5 mm; ML: 0.25 mm; DV: −1.5 mm). Subsequently, a fiber optic ferrule (200 μm O.D., 0.37 NA, Inper, Hangzhou, China) was slowly inserted into the target area to avoid damaging GCaMP6s-expressing neurons. The fiber optic sleeve was firmly fixed to the skull using dental cement (Shanghai New Century Dental Materials Co., Ltd., Shanghai, China, Cat. No. 1520006003) and reinforced with stainless steel screws for stability. Postoperatively, the mice were housed individually in cages and given 10 days of recovery to ensure adequate healing.

### 2.7. Fiber Optic Signal Recording and Analysis

GCaMP6s fluorescence intensities were recorded using fiber photometry (Inper, Hangzhou, China). For each observer group, 7 mice were recorded. Specifically, to record fluorescence signals, a 470 nm LED light (Inper, Hangzhou, China) was reflected by a dichroic mirror (MD498, Thorlabs, Newton, NJ, USA), focused with a ×10 objective lens (NA = 0.3, Olympus, Hachioji, Tokyo) and then coupled to an optical commutator (Inper, Hangzhou, China). An optical fiber (200 μm OD, NA = 0.37, Inper, Hangzhou, China) was used to guide the light between the commutator and im-planted optical fiber. Laser power was adjusted at the tip of the optical fiber to a level of 20–40 μW. Fluorescence was bandpass-filtered (MF525-39, Thorlabs, Newton, NJ, USA) and focused using a ×20 objective lens. Fluorescence signals were collected using a CMOS camera (BASLER, Ahrensburg, Germany). The end of the fiber was imaged at a frame rate of 100. The region-of-interest area size and mean value were set using Inper Signal Software (Version 1.9.3, Inper, Hangzhou, China). The 470 nm and 410 nm light sources were given alternately, and 410 nm was used as the internal control. The de-modulated signal was stored using a sampling frequency of 100 Hz. We used Inper Plot (V1.9.9, Inper) to analyze photometric data. Briefly, we calculated ΔF/F using a baseline (3 s before observer’s head orientation toward demonstrator).

### 2.8. In Vivo Optogenetic Manipulation

For optogenetic inhibition and activation, AAV2/9-CaMKII1α-eNpHR3.0-mcherry (PT-0009, BrainVTA, Wuhan, China) and AAV2/9-CaMKII1α-hChR2-mcherry (PT-0297, BrainVTA, Wuhan, China) were injected into the ACC (AP: 0.5 mm; ML: ±0.3 mm; DV: −1.5 mm). Before behavioral testing, mice were implanted with fiber optic ferrules (200 μm O.D., 0.37 NA, Inper, Hangzhou, China) at the coordinates of AP: 0.5 mm, ML: 0 mm, and DV: −1.4 mm. All fiber optic ferrules were fixed to the skull with bone screws and dental cement. Three weeks later, the mice underwent behavioral testing. All mice were habituated to wearing fiber optic cables before the experiment. In the optogenetic manipulation, the observer mice received continuous light stimulation (ChR2, 470 nm, 20 Hz, 10 mW; eNpHR3.0, 593 nm, continuous, 5 mW). The frequency and duration of light stimulation were controlled through an optogenetic system (ThinkerTech Nanjing Bioscience Inc., Nanjing, China, MGL-F 17061414). In mice injected with mCherry, the same number of laser pulses were applied at the same frequency. The working power of the LED laser output was checked in each experiment to ensure consistency. Six mice in each observer group were manipulated and recorded in both optogenetic actuator experiments.

### 2.9. Statistics

Statistical analyses were performed using GraphPad Prism (version 9.5.0, GraphPad Software, Boston, MA, USA). Sample sizes were not predetermined using statistical methods. Statistical tests used in this study include Student’s t-test, one-way analysis of variance, and two-way analysis of variance. The N used for these analyses represents the number of mice, and n for trial. When parametric tests were used, data normality were confirmed using the Shapiro–Wilk normality test; otherwise using nonparametric tests. Data are reported as mean ± SEM; ns, not significant (*p* > 0.05); * *p* < 0.05, ** *p* < 0.01, *** *p* < 0.001.

## 3. Results

### 3.1. Naïve and Relieved Observers Exhibit Social Preference for a Stressed Demonstrator, Whereas Stressed Observers Lose This Preference

**Figure 1:**
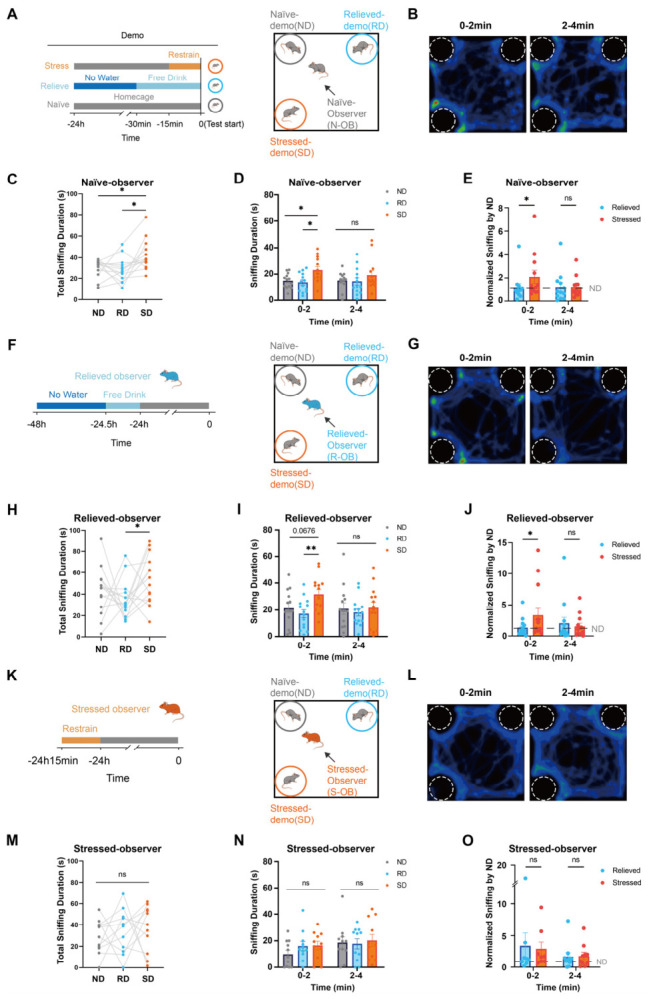
Different emotional experiences have shaped the different social patterns of mice. (**A**) Schematic timeline of experimental procedures for inducing distinct emotional states in demonstrators (Naïve-ND, Relieved-RD, Stressed-SD) and the subsequent social interaction test. (**B**) Representative heatmaps showing the spatial distribution of Naïve-observers (N-OB) during interaction with different demonstrators (Naïve, Relieved, Stressed) in the 0–2 min and 2–4 min intervals. The dashed circles indicate the cages where the demonstrators were placed. (**C**) Total sniffing duration of N-OB toward ND, RD, and SD during the 0–4 min test period (N = 13 mice). (**D**) Temporal analysis of sniffing duration of N-OB across 0–2 min and 2–4 min time bins (N = 13 mice). (**E**) Sniffing duration of N-OB normalized to ND (N = 13 mice). (**F**) Timeline for the induction of the relieved observers (R-OB) prior to the social test (N = 14 mice). (**G**) Representative heatmaps of R-OB interaction patterns. The dashed circles indicate the cages where the demonstrators were placed. (**H**) Total sniffing duration of R-OB toward ND, RD, and SD (N = 14 mice). (**I**) Temporal analysis of sniffing duration of R-OB across time bins (N = 14 mice). (**J**) Sniffing duration of R-OB normalized to the Relieved group (N = 14 mice). (**K**) Timeline for the induction of the stressed state in observers (S-OB) using a restraint stress protocol. (**L**) Representative heatmaps of S-OB interaction patterns. The dashed circles indicate the cages where the demonstrators were placed. (**M**) Total sniffing duration of S-OB toward ND, RD, and SD (N = 11 mice). (**N**) Temporal analysis of sniffing duration of S-OB across time bins (N = 11 mice). (**O**) Sniffing duration of S-OB normalized to the Stressed group (N = 11 mice). Significance levels are denoted as follows: * *p* < 0.05, ** *p* < 0.01. Trends approaching significance are indicated with specific values where *p* < 0.1. Error bars represent SEM.

According the multi-target social interaction test (Figure 1A), we observed that naïve observers exhibited a profound social preference for the stress-demonstrator (SD) over the naïve-demonstrator (ND) and relieved-demonstrator (RD), whereas interaction times between the ND and RD were comparable (Figure 1B,C, detailed statistics in Table S1). Temporal analysis revealed that this preference for the SD was highly acute, peaking during the initial 2 min of the assay and subsequently habituating (Figure 1D,E, detailed statistics in Table S1). This suggests that the 0–2 min window represents a phase of rapid, preferential investigation of stressed conspecifics upon initial exposure to a complex social environment.

We next asked whether the observer’s own internal emotional state modulates this rapid social prioritization. To address this, we utilized relieved or stressed mice as observers in the same paradigm (Figure 1F,K). Relieved observers exhibited behavioral trajectories indistinguishable from naïve mice, displaying a robust innate preference for the SD during the first 2 min (Figure 1G–J, detailed statistics in Table S1). In contrast, stressed mice completely lacked the initial discrimination capacity, interacting equally with all three demonstrators during the 0–4 min phase (Figure 1L–O, detailed statistics in Table S1). Importantly, this loss of social priority was not due to generalized social withdrawal. While relieved mice showed increased total social exploration, naïve and stressed observers exhibited comparable total sniffing durations (Figure S1, detailed statistics in Table S1). Furthermore, recent experiences of stress or relief did not alter anxiety, locomotion, or general sociability in the observers, as measured by the open field and social ability tests (Figures S2 and S3, detailed statistics in Table S1). Consistent with these behavioral findings, there were no significant differences in serum corticosterone levels across the different observer groups (Figure S4, detailed statistics in Table S1). Thus, acute stress specifically impairs social discrimination without broadly suppressing social motivation. Together, these data indicate that a severe negative internal state overrides a mouse’s innate drive to rapidly identify and investigate a distressed peer.

### 3.2. Distinct ACC Neural Dynamics Encode Social Prioritization Depending on the Observer’s Emotional State

**Figure 2:**
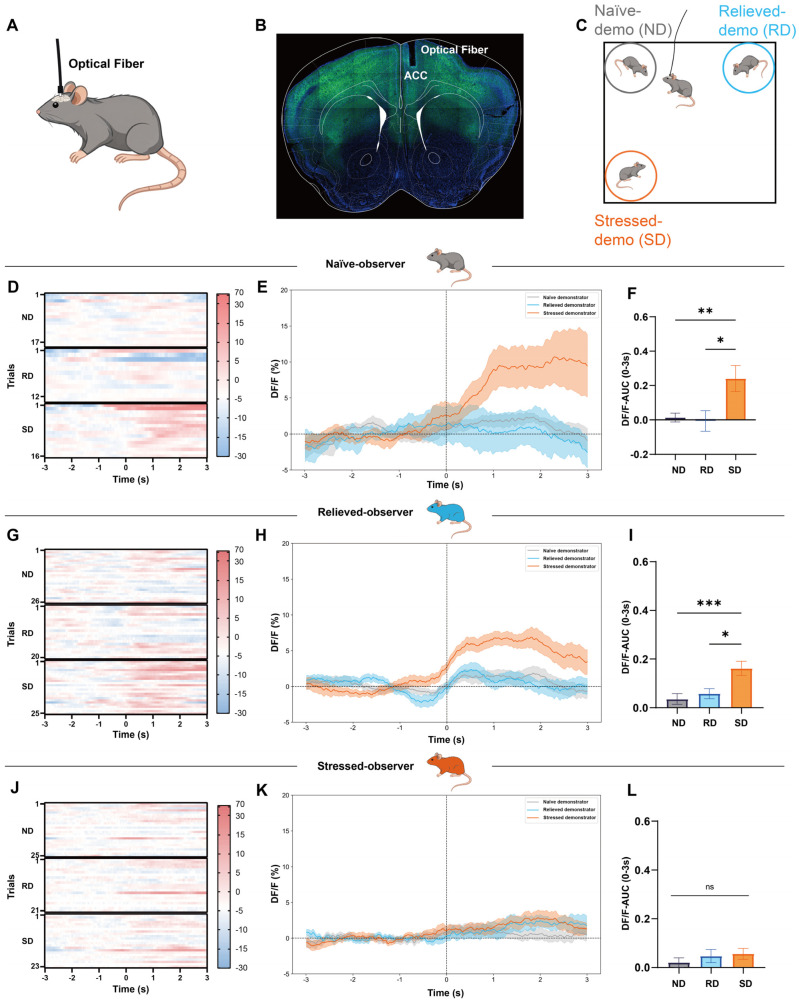
ACC Neural Activity Dynamics During Social Sniffing Across Different Observer Groups (0–2 min period). (**A**) Schematic illustration of the optical fiber implantation targeting the anterior cingulate cortex (ACC) for fiber photometry recording. (**B**) Representative histological image showing the expression of the calcium indicator (green), DAPI (blue), and the placement of the optical fiber tip in the ACC. (**C**) Schematic diagram of the social interaction paradigm where the observer mouse interacts with three types of demonstrators: Naïve demonstrator (ND), Relieved demonstrator (RD), and Stressed demonstrator (SD). (**D**) Heatmaps of calcium signals (ΔF/F) in Naïve observers (N-OB) during interaction with ND, RD, and SD. (**E**) Calcium traces (ΔF/F, Mean ± SEM) of N-OB aligned to the onset of sniffing toward ND, RD, and SD. (**F**) Quantification of the area under the curve (AUC) of ΔF/F signals in N-OB during the 0–3 s window after sniffing onset (N = 7 mice, ND trials n = 17, RD trials n = 12, SD trials n = 16). (**G**) Heatmaps of calcium signals in Relieved observers (R-OB) during interaction with ND, RD, and SD. (**H**) Calcium traces (ΔF/F, Mean ± SEM) of R-OB aligned to the onset of sniffing. (**I**) Quantification of the AUC of ΔF/F signals in R-OB during the 0–3 s window (N = 7 mice, ND trials n = 26, RD trials n = 20, SD trials n = 25). (**J**) Heatmaps of calcium signals in Stressed observers (S-OB) during interaction with ND, RD, and SD. (**K**) Calcium traces (ΔF/F, Mean ± SEM) of S-OB aligned to the onset of sniffing. (**L**) Quantification of the AUC of ΔF/F signals in S-OB during the 0–3 s window (N = 7 mice, ND trials n = 25, RD trials n = 21, SD trials n = 23). Statistical significance is denoted as * *p* < 0.05, ** *p* < 0.01, *** *p* < 0.001, Error bars represent SEM.

To elucidate the neural mechanisms driving these divergent social interaction patterns, we targeted the ACC. As a critical cortical hub for empathy-like behavior, observational pain, and the integration of internal emotional states with external social cues, the ACC emerged as a prime candidate for mediating this rapid social prioritization [17,19,20,21]. We implanted optical fibers into the ACC of Thy1-GCaMP6s transgenic mice [35] and performed *in vivo* fiber photometry during the complex social behavior paradigm (Figure 2A–C).

During the initial 2 min innate discrimination phase, head orientation toward the SD elicited robust ACC Ca^2+^ transients in both naïve and relieved observers (Figure 2D,E,G,H). This neural activation was highly specific to the distressed target, with SD-evoked responses significantly exceeding those triggered by NDs or RDs (Figure 2F,I, detailed statistics in Table S1). Strikingly, this SD-evoked ACC activation was entirely absent in stressed observers (Figure 2L). We further analyzed ACC Ca^2+^ transients during the subsequent 3–4 min habituation phase (Figure S5A,B,D,E,G,H). Aligning with their uniform behavioral interaction during this later window, approaches toward all three demonstrators elicited robust and comparable Ca^2+^ transients in both naïve and relieved observers. In contrast, ACC activity in stressed observers remained persistently blunted, showing no significant elevation (Figure S5C,F,I, detailed statistics in Table S1).

### 3.3. Optogenetic Inhibition of ACC Excitatory Neurons Abolishes Prioritization of Stressed Conspecifics

**Figure 3:**
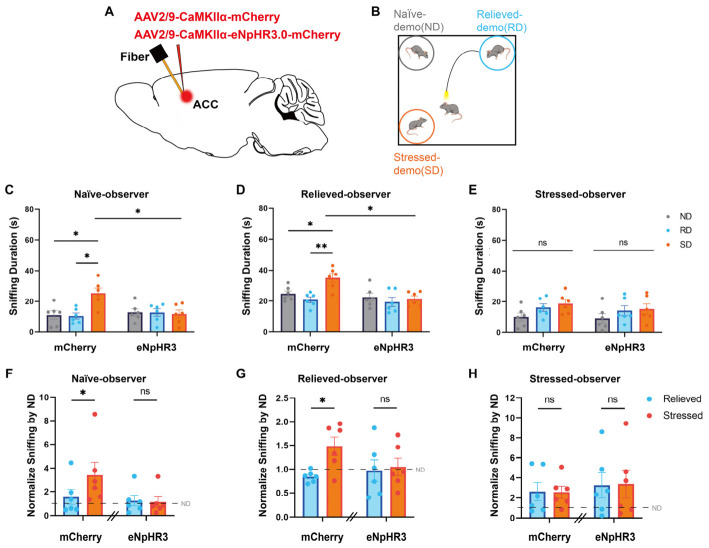
Causal Role of ACC Activity in Modulating Social Preference via Optogenetics. (**A**,**B**) Schematic diagrams illustrating the optogenetic experimental design and viral targeting strategies. (**C**–**E**) Comparative analysis of sniffing duration toward demonstrators in specific emotional states between Control and Experimental groups for Naive, Relieved, and Stressed observers, respectively, under optogenetic manipulation (N = 6 mice). (**F**–**H**) Normalized sniffing time for Relieved and Stressed demonstrator in three observer groups (N = 6 mice). Statistical significance is denoted as * *p* < 0.05 and ** *p* < 0.01. Error bars represent SEM.

Given these correlative neural dynamics, we next sought to determine whether ACC activation is causally required for the rapid social prioritization of stressed conspecifics. We expressed the inhibitory opsin eNpHR3.0, or an mCherry control, in ACC excitatory neurons of observer mice and implanted optical fibers over the injection site (Figure 3A). During the critical 0–2 min window of the complex social test, we delivered continuous yellow light (593 nm, 5 mW) to inhibit these ACC projection neurons (Figure 3B).

Photoinhibition completely abolished the innate social preference for the SD in naïve and relieved observers, reducing their interaction times to levels comparable to the ND and RD (Figure 3C,D,F,G, detailed statistics in Table S1). Notably, optical manipulation in stressed observers yielded no behavioral alteration (Figure 3E,H, detailed statistics in Table S1), confirming that the endogenous ACC engagement required for rapid SD prioritization behavior is already blunted by the animal’s own stressed state.

### 3.4. Optogenetic Activation of the ACC Overrides Internal Stress and Rescues Social Discrimination

**Figure 4:**
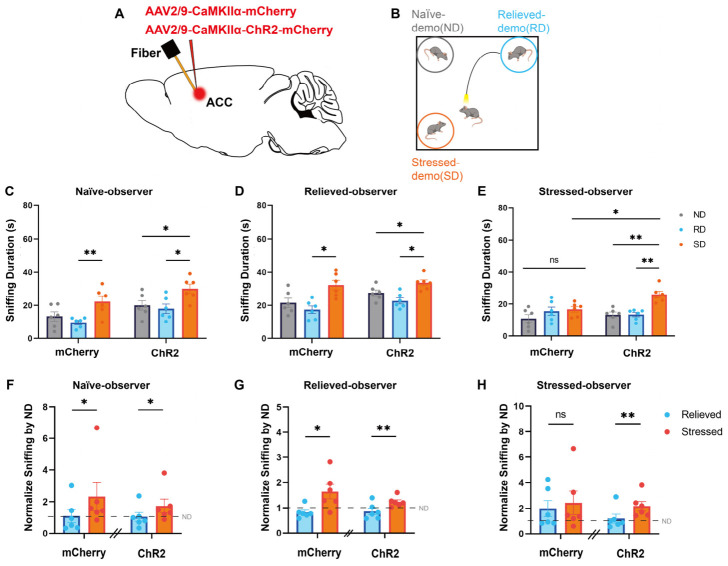
Optogenetic Manipulation of ACC Activity Modulates Social Preference. (**A**,**B**) Schematic diagrams illustrating the optogenetic experimental setup and viral targeting strategies. (**C**–**E**) Comparison of sniffing duration toward demonstrators in a specific emotional state between Control and Experimental groups for Naive, Relieved, and Stressed observers, respectively (N = 6 mice). (**F**–**H**) Normalized sniffing time for Relieved and Stressed demonstrator in three observer groups (N = 6 mice). Statistical significance is denoted as * *p* < 0.05 and ** *p* < 0.01. Error bars represent SEM.

Finally, to test whether ACC activation is sufficient to drive SD prioritization, we expressed ChR2 in ACC excitatory neurons of observer mice to artificially elevate neural activity during the behavioral assay (Figure 4A,B). Photo stimulation of the ACC did not significantly alter the pre-existing social preferences of naïve or relieved observers (Figure 4C,D,F,G, detailed statistics in Table S1), likely because their endogenous ACC responses to the SD were already functioning at an optimal, saturated level.

Crucially, stimulating ACC excitatory neurons in stressed observers restored normal social discrimination, driving a significant increase in interaction time toward the stressed demonstrator (Figure 4E,H, detailed statistics in Table S1).

## 4. Discussion

In this study, we provide a mechanistic framework for how an animal’s internal emotional state dictates innate social prioritization. We demonstrate that, within a complex multi-target environment, naïve mice and mice experiencing emotional relief rapidly prioritize interactions with an acutely stressed conspecific. Conversely, acute stress experience profoundly overrides this innate prosocial drive, abolishing this speedy social discrimination. Using *in vivo* fiber photometry and targeted optogenetic manipulations, we identified excitatory neurons within the ACC as the requisite and manipulable neural substrate gating this state-dependent social discrimination.

### 4.1. State-Dependent Suppression of Multi-Target Prioritization

Naïve and relieved mice exhibit a rapid preference for distressed conspecifics, whereas prior restraint stress completely abolishes this early-stage discrimination. Our control data confirm this deficit is not driven by acute physiological arousal or reduced general social motivation. Previous binary-choice studies have shown that prior negative experiences can abolish prosocial approaches toward peers with similar experiences [11]. Our findings align with this stress-induced loss of preference, but fundamentally extend the framework to a complex social landscape. By presenting three distinct emotional valences simultaneously, we demonstrate that prior stress induces a profound failure to filter and prioritize competing social signals. Our fiber photometry data establish the ACC as the specific neural substrate driving this multi-target prioritization. We propose that severe stress selectively blunts ACC responsiveness; consequently, despite retaining basic social motivation, the observer loses the neural capacity to resolve complex social conflicts and prioritize distressed targets.

### 4.2. The ACC Acts as a State-Dependent Gatekeeper for Empathy-Like Behavior

While the ACC is a well-established core node of the limbic system involved in processing self-experienced pain and negative emotion [36,37,38], our findings significantly expand its functional repertoire in social contexts. Beyond basic affective processing, the ACC is widely recognized as a critical cortical center for broader executive functions, including conflict resolution, cognitive control, and cost–benefit decision-making [16,39,40]. Deploying prosocial behavior in a dynamic, multi-target environment inherently requires the integration of these affective and cognitive streams, as an animal must continuously weigh the emotional valence of external distress cues against its own internal state and survival needs. Our data position the ACC as the critical integrative node where these computations converge. We demonstrate that ACC population activity is robustly tuned to the observation of conspecific distress, but this neural encoding is heavily gated by the observer’s emotional history. Through bidirectional optogenetic manipulations, we establish that the ACC does not merely act as a passive sensory processor. Rather, it utilizes these broader executive capacities to evaluate external distress cues relative to the animal’s internal affective state, dynamically orchestrating the decision to approach or avoid.

## 5. Conclusions

By integrating the observer’s internal emotional history into a multi-target social framework, this study provides a neurobiological foundation for how complex social decisions are governed by internal states. While our findings establish the ACC as the critical cortical node for this process, future research must delineate the specific downstream projection targets of these ACC ensembles to fully map this state-dependent circuitry. Ultimately, our findings build upon recent behavioral models to highlight the ACC as a dynamic gatekeeper, continuously weighing an animal’s internal emotional state against external environmental demands to orchestrate multi-target social prioritization.

## Data Availability

The raw data supporting the conclusions of this article will be made available by the authors on request.

## Supplementary Materials

The following supporting information can be downloaded at https://www.mdpi.com/article/10.3390/brainsci16060658/s1. Figure S1: Total social interaction time across different observer groups (0–4 min); Figure S2: Locomotor activity and anxiety-like behavior in the open field test across different observer groups.; Figure S3: Social behavior assessment in the three-chamber test across different observer groups ; Figure S4: Serum corticosterone levels across different observer groups; Figure S5: ACC Neural Activity Dynamics During Social Sniffing Across Different Observer Groups (2–4 min period); Table S1: Detailed statistical information.

## Abbreviations

The following abbreviations are used in this manuscript:

ACC	Anterior cingulate cortex
ND	Naïve demonstrator
RD	Relieved demonstrator
SD	Stressed demonstrator
NOB	Naïve observer
ROB	Relieved observer
SOB	Stressed observer

## References

[1] Fang S., Luo Z., Wei Z., Qin Y., Zheng J., Zhang H., Jin J., Li J., Miao C., Yang S. (2024). Sexually Dimorphic Control of Affective State Processing and Empathic Behaviors. Neuron.

[2] Smith M.L., Asada N., Malenka R.C. (2021). Anterior Cingulate Inputs to Nucleus Accumbens Control the Social Transfer of Pain and Analgesia. Science.

[3] Ueno H., Suemitsu S., Murakami S., Kitamura N., Wani K., Okamoto M., Matsumoto Y., Aoki S., Ishihara T. (2018). Empathic Behavior According to the State of Others in Mice. Brain Behav..

[4] Decety J., Bartal I.B.-A., Uzefovsky F., Knafo-Noam A. (2016). Empathy as a Driver of Prosocial Behaviour: Highly Conserved Neurobehavioural Mechanisms across Species. Philos. Trans. R. Soc. Lond. B Biol. Sci..

[5] Sivaselvachandran S., Acland E.L., Abdallah S., Martin L.J. (2018). Behavioral and Mechanistic Insight into Rodent Empathy. Neurosci. Biobehav. Rev..

[6] Scheggia D., Managò F., Maltese F., Bruni S., Nigro M., Dautan D., Latuske P., Contarini G., Gomez-Gonzalo M., Requie L.M. (2020). Somatostatin Interneurons in the Prefrontal Cortex Control Affective State Discrimination in Mice. Nat. Neurosci..

[7] Ferretti V., Maltese F., Contarini G., Nigro M., Bonavia A., Huang H., Gigliucci V., Morelli G., Scheggia D., Managò F. (2019). Oxytocin Signaling in the Central Amygdala Modulates Emotion Discrimination in Mice. Curr. Biol..

[8] Kaidanovich-Beilin O., Lipina T., Vukobradovic I., Roder J., Woodgett J.R. (2011). Assessment of Social Interaction Behaviors. J. Vis. Exp..

[9] Arakawa H. (2023). Revisiting Sociability: Factors Facilitating Approach and Avoidance During the Three-Chamber Test. Physiol. Behav..

[10] Hanzel M., Fernando K., Maloney S.E., Horn Z., Gong S., Mätlik K., Zhao J., Pasolli H.A., Heissel S., Dougherty J.D. (2024). Mice Lacking Astn2 Have ASD-like Behaviors and Altered Cerebellar Circuit Properties. Proc. Natl. Acad. Sci. USA.

[11] Maltese F., Pacinelli G., Monai A., Bernardi F., Capaz A.M., Niello M., Walle R., de Leon N., Managò F., Leroy F. (2025). Self-Experience of a Negative Event Alters Responses to Others in Similar States through Prefrontal Cortex CRF Mechanisms. Nat. Neurosci..

[12] Israelashvili J., Sauter D.A., Fischer A.H. (2020). Different Faces of Empathy: Feelings of Similarity Disrupt Recognition of Negative Emotions. J. Exp. Soc. Psychol..

[13] Völlm B.A., Taylor A.N., Richardson P., Corcoran R., Stirling J., McKie S., Deakin J.F., Elliott R. (2006). Neuronal Correlates of Theory of Mind and Empathy: A Functional Magnetic Resonance Imaging Study in a Nonverbal Task. NeuroImage.

[14] Carr L., Iacoboni M., Dubeau M.C., Mazziotta J.C., Lenzi G.L. (2003). Neural Mechanisms of Empathy in Humans: A Relay from Neural Systems for Imitation to Limbic Areas. Proc. Natl. Acad. Sci. USA.

[15] Lamm C., Batson C.D., Decety J. (2007). The Neural Substrate of Human Empathy: Effects of Perspective-Taking and Cognitive Appraisal. J. Cogn. Neurosci..

[16] Monosov I.E., Haber S.N., Leuthardt E.C., Jezzini A. (2020). Anterior Cingulate Cortex and the Control of Dynamic Behavior in Primates. Curr. Biol..

[17] Shackman A.J., Salomons T.V., Slagter H.A., Fox A.S., Winter J.J. (2011). The Integration of Negative Affect, Pain and Cognitive Control in the Cingulate Cortex. Nat. Rev. Neurosci..

[18] Singer T., Seymour B., O’Doherty J., Kaube H., Dolan R.J., Frith C.D. (2004). Empathy for Pain Involves the Affective but Not Sensory Components of Pain. Science.

[19] Etkin A., Egner T., Kalisch R. (2011). Emotional Processing in Anterior Cingulate and Medial Prefrontal Cortex. Trends Cogn. Sci..

[20] Stevens F.L., Hurley R.A., Taber K.H. (2011). Anterior Cingulate Cortex: Unique Role in Cognition and Emotion. J. Neuropsychiatry Clin. Neurosci..

[21] Allman J.M., Hakeem A., Erwin J.M., Nimchinsky E., Hof P. (2001). The Anterior Cingulate Cortex: The Evolution of an Interface Between Emotion and Cognition. Unity of Knowledge: The Convergence of Natural and Human Science.

[22] Klein A.S., Gogolla N. (2021). How Mice Feel Each Other’s Pain or Fear. Science.

[23] Guo B., Chen J., Chen Q., Ren K., Feng D., Mao H., Yao H., Yang J., Liu H., Liu Y. (2019). Anterior Cingulate Cortex Dysfunction Underlies Social Deficits in Shank3 Mutant Mice. Nat. Neurosci..

[24] Song Q., Wei A., Xu H., Gu Y., Jiang Y., Dong N., Zheng C., Wang Q., Gao M., Sun S. (2024). An ACC-VTA-ACC Positive-Feedback Loop Mediates the Persistence of Neuropathic Pain and Emotional Consequences. Nat. Neurosci..

[25] Olsson A., Nearing K.I., Phelps E.A. (2007). Learning Fears by Observing Others: The Neural Systems of Social Fear Transmission. Soc. Cogn. Affect. Neurosci..

[26] Olsson A., Phelps E.A. (2007). Social Learning of Fear. Nat. Neurosci..

[27] Zhou C., Zhou Z., Han Y., Lei Z., Li L., Montardy Q., Liu X., Xu F., Wang L. (2018). Activation of Parvalbumin Interneurons in Anterior Cingulate Cortex Impairs Observational Fear. Sci. Bull..

[28] Choi J., Lee Y.-B., So D., Kim J.Y., Choi S., Kim S., Keum S. (2025). Cortical Representations of Affective Pain Shape Empathic Fear in Male Mice. Nat. Commun..

[29] Keum S., Shin H.-S. (2019). Neural Basis of Observational Fear Learning: A Potential Model of Affective Empathy. Neuron.

[30] Jabarin R., Mohapatra A.N., Ray N., Netser S., Wagner S. (2025). Distinct Prelimbic Cortex Neuronal Responses to Emotional States of Others Drive Emotion Recognition in Adult Mice. Curr. Biol..

[31] Kielbinski M., Bernacka J. (2024). Fiber Photometry in Neuroscience Research: Principles, Applications, and Future Directions. Pharmacol. Rep..

[32] Simpson E.H., Akam T., Patriarchi T., Blanco-Pozo M., Burgeno L.M., Mohebi A., Cragg S.J., Walton M.E. (2024). Lights, Fiber, Action! A Primer on in Vivo Fiber Photometry. Neuron.

[33] Legaria A.A., Matikainen-Ankney B.A., Yang B., Ahanonu B., Licholai J.A., Parker J.G., Kravitz A.V. (2022). Fiber Photometry in Striatum Reflects Primarily Nonsomatic Changes in Calcium. Nat. Neurosci..

[34] Boyden E.S., Zhang F., Bamberg E., Nagel G., Deisseroth K. (2005). Millisecond-Timescale, Genetically Targeted Optical Control of Neural Activity. Nat. Neurosci..

[35] Dana H., Chen T.-W., Hu A., Shields B.C., Guo C., Looger L.L., Kim D.S., Svoboda K. (2014). Thy1-GCaMP6 Transgenic Mice for Neuronal Population Imaging In Vivo. PLoS ONE.

[36] Zhou Z., Gao Y., Bao W., Liang K., Cao L., Tang M., Li H., Hu X., Zhang L., Sun H. (2024). Distinctive Intrinsic Functional Connectivity Alterations of Anterior Cingulate Cortex Subdivisions in Major Depressive Disorder: A Systematic Review and Meta-Analysis. Neurosci. Biobehav. Rev..

[37] Bush G., Luu P., Posner M.I. (2000). Cognitive and Emotional Influences in Anterior Cingulate Cortex. Trends Cogn. Sci..

[38] LaGraize S.C., Labuda C.J., Rutledge M.A., Jackson R.L., Fuchs P.N. (2004). Differential Effect of Anterior Cingulate Cortex Lesion on Mechanical Hypersensitivity and Escape/Avoidance Behavior in an Animal Model of Neuropathic Pain. Exp. Neurol..

[39] Tervo D.G.R., Kuleshova E., Manakov M., Proskurin M., Karlsson M., Lustig A., Behnam R., Karpova A.Y. (2021). The Anterior Cingulate Cortex Directs Exploration of Alternative Strategies. Neuron.

[40] Brockett A.T., Tennyson S.S., deBettencourt C.A., Gaye F., Roesch M.R. (2020). Anterior Cingulate Cortex Is Necessary for Adaptation of Action Plans. Proc. Natl. Acad. Sci. USA.

