# Supplementary material for: Anterior Cingulate Cortex Mediates State-Dependent Prioritization of Distressed Conspecifics
Source: Brain Sci. 2026 Jun 22;16(6):658. doi: 10.3390/brainsci16060658 (PMC13297314; doi:10.3390/brainsci16060658)
Supplement: Supplementary file 1 [file brainsci-16-00658-s001.zip › brainsci-4310172-Supplementary Materials.pdf]

Supplementary Materials:

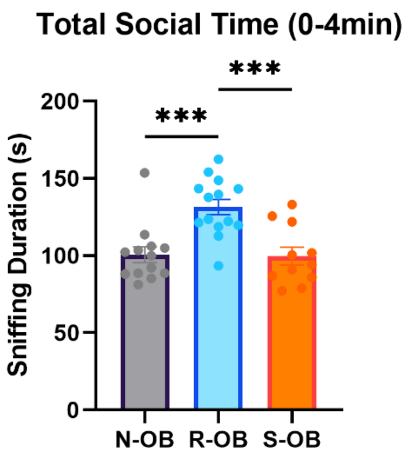

Figure S1. Total Social Interaction Time Across Different Observer Groups (0–4 min). Statistical significance is denoted as ***p < 0.001. Error bars represent SEM.

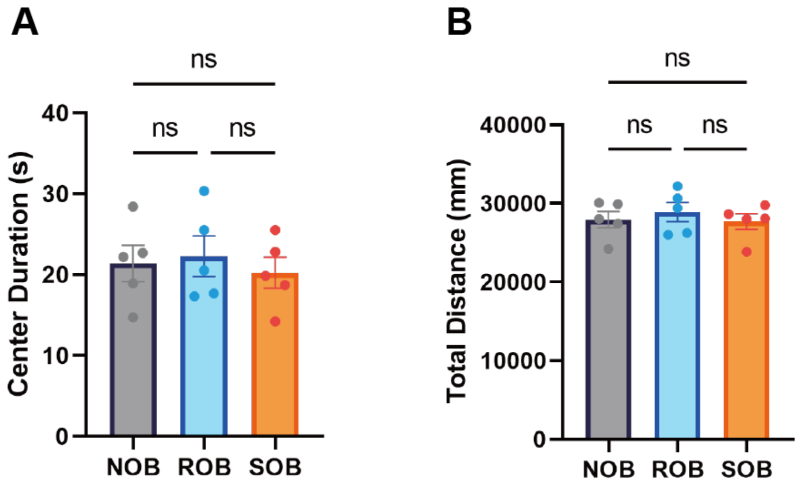

Figure S2. Locomotor activity and anxiety-like behavior in the open field test across different observer groups. **(A)** Time spent in the center zone of the open field arena. (N = 5 mice) **(B)** Total distance traveled during the test session. (N = 5 mice). Error bars represent SEM.

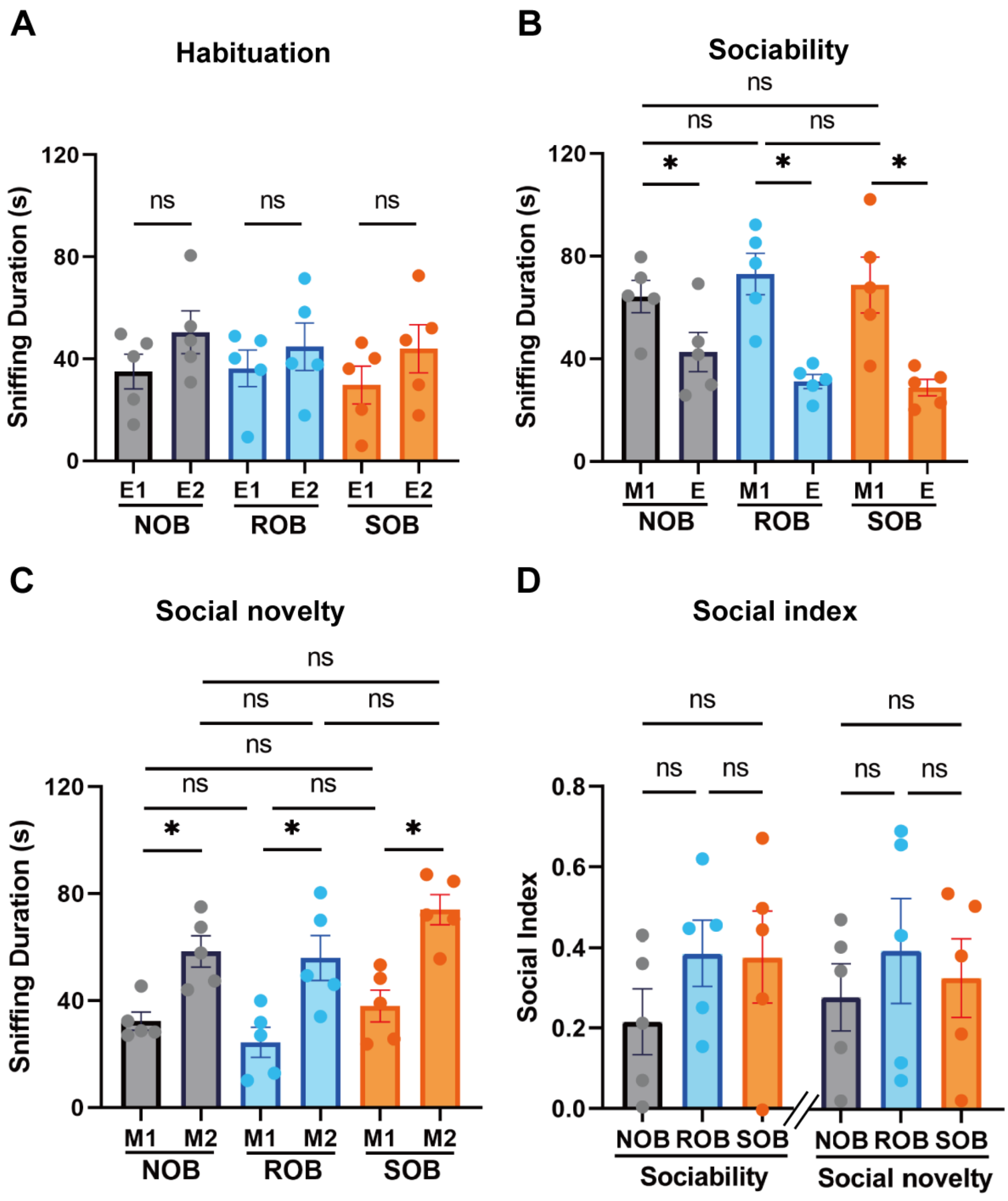

Figure S3. Social behavior assessment in the three-chamber test across different observer groups. **(A)** Sniffing time directed towards two empty metal wires cages (E1 and E2) during the habituation phase to assess baseline exploration preference. (N = 5 mice) **(B)** Sniffing time spent exploring an empty metal wires cage (E) versus a stranger mouse (M1) during the sociability phase. (N = 5 mice) **(C)** Sniffing time spent exploring a familiar mouse (M1) versus a second stranger mouse (M2) during the social novelty phase. (N = 5 mice) **(D)** Social preference index calculated for both the sociability and social novelty phases to quantify interaction bias. (N = 5 mice) Statistical significance is denoted as *$p < 0.05$. Error bars represent SEM.

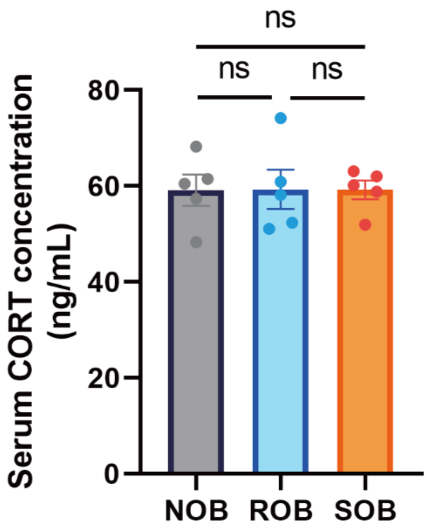

Figure S4. Serum corticosterone levels across different observer groups. (N = 5 mice) Error bars represent SEM.

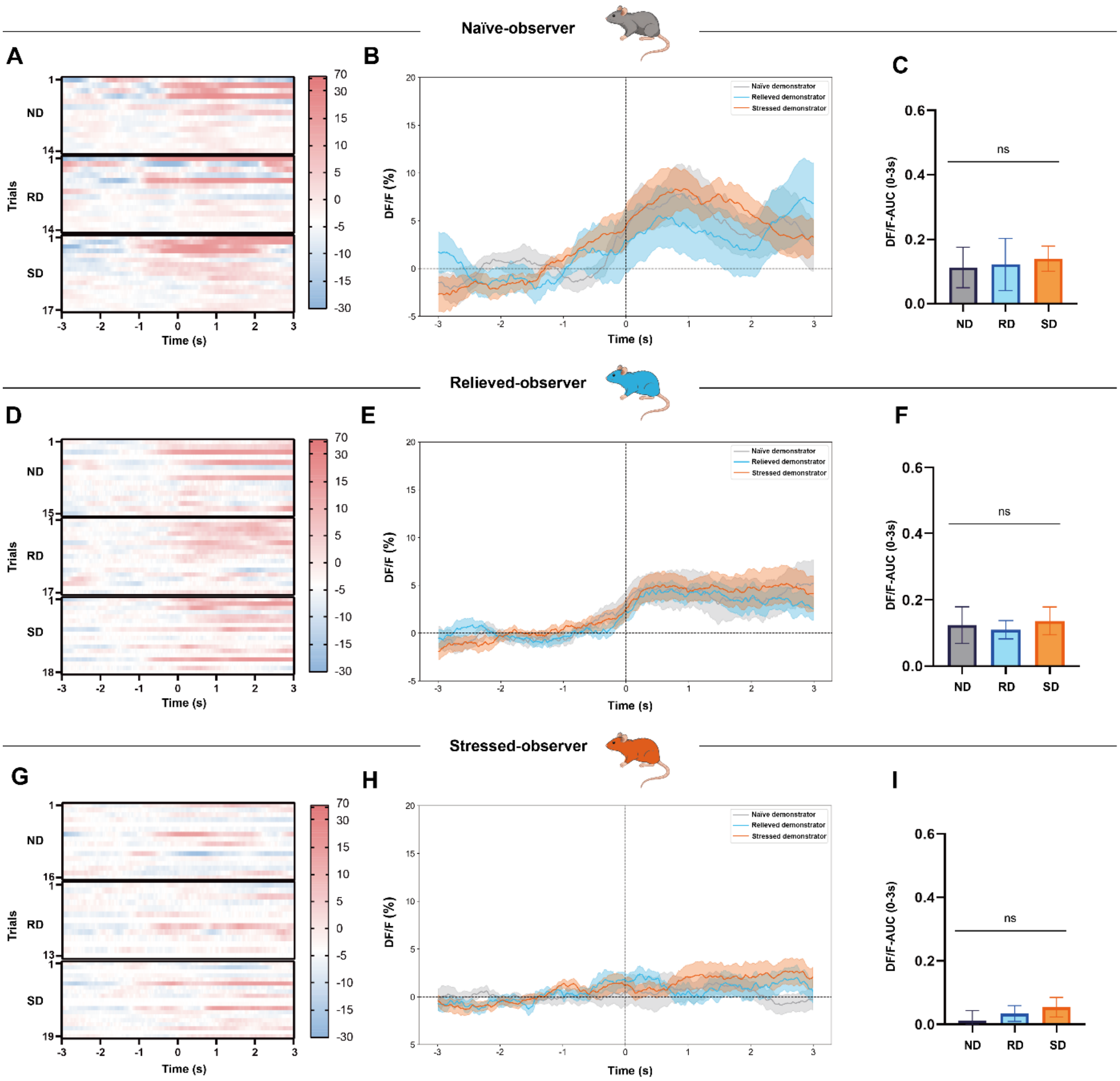

Figure S5. ACC Neural Activity Dynamics During Social Sniffing Across Different Observer Groups (2–4 min period). Error bars represent SEM. (A) Heatmaps of calcium signals (ΔF/F) in Naïve observers (N-OB) during interaction with ND, RD, and SD. (B) Average calcium traces (ΔF/F) of N-OB aligned to the onset of sniffing toward ND, RD, and SD. (C) Quantification of the area under the curve (AUC) of ΔF/F signals in N-OB during the 0–3 s window after sniffing onset. (N = 7 mice, ND trials n = 14, RD trials n = 14, SD trials n = 17) (D) Heatmaps of calcium signals in Relieved observers (R-OB) during interaction with ND, RD, and SD. (E) Average calcium traces of R-OB aligned to the onset of sniffing. (F) Quantification of the AUC of ΔF/F signals in R-OB during the 0–3 s window. (N = 7 mice, ND trials n = 15, RD trials n = 17, SD trials n = 18) (G) Heatmaps of calcium signals in Stressed observers (S-OB) during interaction with ND, RD, and SD. (H) Average calcium traces of S-OB aligned to the onset of sniffing. (I) Quantification of the AUC of ΔF/F signals in S-OB during the 0–3 s window. (N = 7 mice, ND trials n = 16, RD trials n = 13, SD trials n = 19). Error bars represent SEM.
